# Editorial of Special Issue: The Toxicity of Nanomaterials and Legacy Contaminants: Risks to the Environment and Human Health

**DOI:** 10.3390/ijms241411723

**Published:** 2023-07-21

**Authors:** Ana Teresa Reis, Carla Costa, Sónia Fraga

**Affiliations:** 1 Environmental Health Department, National Institute of Health Dr. Ricardo Jorge, 4000-055 Porto, Portugal; carla.trindade@insa.min-saude.pt (C.C.); sonia.fraga@insa.min-saude.pt (S.F.); 2 EPIUnit—Instituto de Saúde Pública, Universidade do Porto, 4050-600 Porto, Portugal; 3 Laboratório Para a Investigação Integrativa e Translacional em Saúde Populacional (ITR), Universidade do Porto, 4050-600 Porto, Portugal

Nanotechnology and the incorporation of nanomaterials (NM) into everyday products help to solve problems in society and improve the quality of life, allowing for major advances in the technological, industrial, and medical fields. Despite this positive and encouraging side of nanotechnology, the potential risks of NM to human health and the environment, as well as the ethical, legal, and social implications associated with nanotechnology, cannot be disregarded. Indeed, the same characteristics that make NM interesting from a technological application point of view may be undesirable upon their release into the environment. In fact, hundreds of tons of NM are released into the environment every year. The reduced dimensions of NM facilitate their diffusion into and transport through the atmosphere, water, and soil, and as well as their uptake and (bio)accumulation in organisms [1,2].

Nanotoxicology has emerged as a discipline that seeks to assess the potential risk of NM, integrating knowledge and resources from material science, biology, toxicology, and analytical chemistry. Several studies have alerted us to the risks that certain NM represent for the environment and for our health, depending on their persistence and circulation in ecosystems, on the dose and responses of organisms to acute and chronic exposure to these substances, and on the ability of organisms to (bio)accumulate and/or excrete them. However, knowledge of the harmful effects of these contaminants of emerging concern is still insufficient, including mixture effects [2,3,4]. Efforts to advance our knowledge on the reactivity of NM and their effects have been made using mostly in vitro and in vivo models; however, in recent years, in silico approaches and quantitative structure–activity relationship (QSAR) modeling have been gaining more attention [5]. Nanotoxicity assessment using in vitro models gathers important information regarding the mechanism(s) of action of NM at the cellular and molecular levels. These models also offer the benefits of reduced costs and ethical concerns over animal welfare (3Rs principle), usually resulting in the faster toxicity screening of chemicals, an advantage considering the increasing number of materials and contaminant combinations to be tested. However, they lack the complexity and metabolic capabilities that in vivo models provide, which is important in identifying the relationship between exposure dose and the occurrence of adverse effects, and in understanding how the body handles NM in terms of their absorption, distribution, metabolism, and excretion (ADME) [6].

Some of the aspects mentioned above have been explored in the papers included in this Special Issue.

Inhalation is one of the main pathways for xenobiotics to penetrate the human body and, consequently, the respiratory system is one of the most studied targets of the infiltration of nanomaterials [6]. In this regard, Bessa et al. [7] addressed the effects of incidental fine and nanosized airborne particles emitted during the industrial thermal spray coating processes, in particular high velocity oxy fuel (HVOF) and atmospheric plasma spraying (APS). Overall, particles derived from both processes were found to be toxic to human alveolar epithelial cells, though different mechanisms were involved in the induced responses. HVOF particles were more cytotoxic compared to APS particles, most likely due to differences in their elemental composition. However, particles derived from both processes caused DNA damage, with APS particles increasing the levels of H2AX phosphorylation, while HVOF particles caused 8-oxo-7,8-dihydro-20-deoxyguanosine (8-oxo-dG) oxidative DNA lesions, with the effect being more evident at lower concentrations of the nanosized particles compared to the fine particles. This study highlights that workers from industries employing processes with a high potential for (nano)particle release may be at (increased) risk of adverse health effects, and therefore must be closely monitored in order to guarantee their health and safety.

Ziglari et al. [8] also focused on the respiratory system, studying the mechanism of lysosomal membrane permeabilization triggered by NM in murine alveolar macrophages. Using a quick non-invasive method to directly measure lysosomal membrane potential, which was specifically developed for this study, the authors showed that nanoparticles (ZnO and TiO_2_) and crystalline SiO_2_ increase lysosomal permeability to cations (potassium), leading to lysosomal membrane hyperpolarization that may potentially damage the lysosomal membrane. The authors also explained that hyperpolarization must exceed 40 mV to cause lysosomal membrane permeabilization. These results provide additional insight into the possible role of particle-induced membrane hyperpolarization on lysosomal membrane permeabilization and other cascade events, such as NLRP3 activation and cell death.

One major knowledge gap that has been identified is the effect of concomitant exposure to NM with other contaminants. It is expected that, when released into the environment, NM will coexist and interact with other potentially toxic species. Although scarce, existing data in the literature indicate that these interactions can affect the physicochemical characteristics of NM, as well as their toxicity and absorption by cells/organisms [9,10]. Naasz and colleagues [11] reviewed the available data from 151 studies on NM–chemical mixtures in environmental organisms and concluded that NM may modify the effects of chemicals on organisms in various ways.

Rosário et al. [12] studied and discussed the differences resulting from the exposure of liver and neuronal cells to single nanomaterials (TiO_2_ and CeO_2_) and potentially toxic elements (As and Hg) or binary mixtures of these compounds. The authors addressed the viability and proliferative capacity of the cells, as well as changes in their cell cycle. Compared to single exposures, and depending on the mixture, the concentration, the exposure time, and the cell, they observed: (1) no change in toxicological response; (2) potentiation effects, particularly after long-term exposure, proving that it is extremely important to address chronic effects, especially if chemicals with a long half-life in humans are present in the mixture; and (3) antagonistic effects on specific conditions, such as the formation of large NP aggregates, that, in turn, hinder their uptake by the cells, block the other contaminants’ access to the cell, and/or if the NP (e.g., CeO_2_NPs) has the ability to act as an antioxidant and reduce oxidative stress in the cell.

This complexity of responses after co-exposure to two compounds was also observed by Carvalhais et al. [13]. These authors studied the isolated and combined effects of two UV filters, namely TiO_2_NPs and oxybenzone, conducting in vivo experiments on *Scophthalmus maximus* (turbot). Simultaneous exposure to both UV filters resulted in either favorable or unfavorable outcomes, depending on the organ, parameter, and post-exposure time.

Even though the respiratory system is one the main direct targets (the gastro-intestinal system is also considered one in the case of ingestion), NM can reach the circulatory system, cross blood–organ barriers, and cause toxicity to other organs [14]. Nanoparticle exposure has been associated with an elevated risk of cardiovascular dysfunction [15,16]. Klinova et al. [17] investigated in their well-designed study the single and mixture toxicity of lead (PbO) and cadmium oxide (CdO) NP, with a focus on the cardiac inotropic effects, following repeated intraperitoneal administration in rats. Using a combination of classical biochemical, physiological, and pharmacological methods, these authors demonstrated that both NP, particularly when administered as a mixture, induced marked toxicity as evidenced by cardiac, hepatic, and renal morphometric changes, increased DNA fragmentation in blood nucleated cells, decreased sliding velocity of myofilaments, and changes upon right ventricle trabeculae and papillary muscles’ mechanical work, most likely associated with alterations in intracellular calcium kinetics.

Finally, the lack of harmonized protocols for nanotoxicity testing has been highlighted [18], and it has been shown that NP themselves may interfere with biological assays [19], leading to inaccurate results and hindering developments in nanotoxicology. In this context, and considering the existing evidence that cytochalasin-B, used in in vitro cytokinesis-block micronucleus (CBMN), affects the uptake of nanomaterials, hindering its use in the genotoxicity testing of nanomaterials. Fernández-Bertólez et al. [20] tested TiO_2_NP uptake and induced genotoxicity in neuroblastoma (SH-SY5Y) cells using flow cytometry and CBMN, after three different treatment options with cytochalasin-B, as recommended by the Organisation for Economic Co-operation and Development (OECD). The results showed that, even though there was no interference of cytochalasin-B in the uptake, micronuclei induction was affected by the presence of cytochalasin-B in the medium, possibly causing false positive results. Once again, it becomes clear that the identification and establishment of alternative protocols for the assessment of nanomaterials’ genotoxicity are urgently required.

The works presented in this Special Issue are intended to advance our knowledge on NM toxicity. Both occupational and environmental exposure were considered, and the toxic potential in several target organs (lungs, brain, liver, heart, and intestine) as well as multiple biomarkers were measured through in vitro and in vivo experiments. Some important demands in the field of nanotoxicology have not been adequately met, such as the co-exposure of NM and other contaminants and the optimization of protocols to conduct toxicity assessments of NPs, highlighting the need for more studies dedicated to this complex issue in future. Altogether, the results presented by the contributing authors reinforce the evidence base for preventive actions and the development of safe-by-design NM, while also furthering the analytical progress of new methodologies to assess the toxicity of pollutants, either as single contaminants or in mixtures. Overall, this Special Issue offers scientific evidence and background for researchers working in the field of epidemiology, toxicology, biochemistry, and nanomedicine.

## References

[1] Yang W., Wang L., Mettenbrink E.M., DeAngelis P.L., Wilhelm S. (2021). Nanoparticle Toxicology. Annu. Rev. Pharmacol. Toxicol..

[2] Bundschuh M., Filser J., Lüderwald S., McKee M.S., Metreveli G., Schaumann G.E., Schulz R., Wagner S. (2018). Nanoparticles in the Environment: Where Do We Come from, Where Do We Go To?. Environ. Sci. Eur..

[3] Sengul A.B., Asmatulu E. (2020). Toxicity of Metal and Metal Oxide Nanoparticles: A Review. Environ. Chem. Lett..

[4] Saleh T.A. (2020). Nanomaterials: Classification, Properties, and Environmental Toxicities. Environ. Technol. Innov..

[5] Huang H.J., Lee Y.H., Hsu Y.H., Liao C.T., Lin Y.F., Chiu H.W. (2021). Current Strategies in Assessment of Nanotoxicity: Alternatives to in Vivo Animal Testing. Int. J. Mol. Sci..

[6] Bessa M.J., Brandão F., Rosário F., Moreira L., Reis A.T., Valdiglesias V., Laffon B., Fraga S., Teixeira J.P. (2023). Assessing the in Vitro Toxicity of Airborne (Nano)Particles to the Human Respiratory System: From Basic to Advanced Models. J. Toxicol. Environ. Health B Crit. Rev..

[7] Bessa M.J., Brandão F., Fokkens P.H.B., Leseman D.L.A.C., Boere A.J.F., Cassee F.R., Salmatonidis A., Viana M., Monfort E., Fraga S. (2022). Unveiling the Toxicity of Fine and Nano-Sized Airborne Particles Generated from Industrial Thermal Spraying Processes in Human Alveolar Epithelial Cells. Int. J. Mol. Sci..

[8] Ziglari T., Wang Z., Holian A. (2021). Contribution of Particle-Induced Lysosomal Membrane Hyperpolarization to Lysosomal Membrane Permeabilization. Int. J. Mol. Sci..

[9] Zhang F., Wang Z., Peijnenburg W.J.G.M., Vijver M.G. (2022). Review and Prospects on the Ecotoxicity of Mixtures of Nanoparticles and Hybrid Nanomaterials. Environ. Sci. Technol..

[10] Deng R., Lin D., Zhu L., Majumdar S., White J.C., Gardea-Torresdey J.L., Xing B. (2017). Nanoparticle Interactions with Co-Existing Contaminants: Joint Toxicity, Bioaccumulation and Risk. Nanotoxicology.

[11] Naasz S., Altenburger R., Kühnel D. (2018). Environmental Mixtures of Nanomaterials and Chemicals: The Trojan-Horse Phenomenon and Its Relevance for Ecotoxicity. Sci. Total Environ..

[12] Rosário F., Costa C., Lopes C.B., Estrada A.C., Tavares D.S., Pereira E., Teixeira J.P., Reis A.T. (2022). In Vitro Hepatotoxic and Neurotoxic Effects of Titanium and Cerium Dioxide Nanoparticles, Arsenic and Mercury Co-Exposure. Int. J. Mol. Sci..

[13] Carvalhais A., Pereira B., Sabato M., Seixas R., Dolbeth M., Marques Guilherme A.S., Pereira P., Pacheco M., Mieiro C. (2021). Mild Effects of Sunscreen Agents on a Marine Flatfish: Oxidative Stress, Energetic Profiles, Neurotoxicity and Behaviour in Response to Titanium Dioxide Nanoparticles and Oxybenzone. Int. J. Mol. Sci..

[14] Raftis J.B., Miller M.R. (2019). Nanoparticle Translocation and Multi-Organ Toxicity: A Particularly Small Problem. Nano Today.

[15] Cao Y., Gong Y., Liao W., Luo Y., Wu C., Wang M., Yang Q. (2018). A Review of Cardiovascular Toxicity of TiO_2_, ZnO and Ag Nanoparticles (NPs). BioMetals.

[16] Yu X., Hong F., Zhang Y.Q. (2016). Bio-Effect of Nanoparticles in the Cardiovascular System. J. Biomed. Mater. Res. A.

[17] Klinova S.V., Katsnelson B.A., Minigalieva I.A., Gerzen O.P., Balakin A.A., Lisin R.V., Butova K.A., Nabiev S.R., Lookin O.N., Katsnelson L.B. (2021). Cardioinotropic Effects in Subchronic Intoxication of Rats with Lead and/or Cadmium Oxide Nanoparticles. Int. J. Mol. Sci..

[18] Krug H.F., Nau K. (2022). Editorial: Methods and Protocols in Nanotoxicology. Front. Toxicol..

[19] Ong K.J., MacCormack T.J., Clark R.J., Ede J.D., Ortega V.A., Felix L.C., Dang M.K.M., Ma G., Fenniri H., Veinot J.G.C. (2014). Widespread Nanoparticle-Assay Interference: Implications for Nanotoxicity Testing. PLoS ONE.

[20] Fernández-Bertólez N., Brandão F., Costa C., Pásaro E., Teixeira J.P., Laffon B., Valdiglesias V. (2021). Suitability of the in Vitro Cytokinesis-Block Micronucleus Test for Genotoxicity Assessment of TiO_2_ Nanoparticles on SH-SY5Y Cells. Int. J. Mol. Sci..

